# Hemodynamic, neurological, and cognitive outcomes after surgical revascularisation in occlusive cerebrovascular disease: Study protocol for single-arm interventional cohort study

**DOI:** 10.1371/journal.pone.0354103

**Published:** 2026-08-07

**Authors:** Chingiz Nurimanov, Karashash Menlibayeva, Iroda Mammadinova, Ainur Turzhanova, Seitzhan Aidarov, Daultay Batyrkhanov, Assel Kabykenova, Yerbol Makhambetov, Assylbek Kaliyev

**Affiliations:** 1 Department of Vascular and Functional Neurosurgery, National Centre for Neurosurgery, Astana, Kazakhstan; 2 Department of Population Health Sciences, King’s College London, London, United Kingdom; 3 Scientific Research Management Department, National Centre for Neurosurgery, Astana, Kazakhstan; 4 Department of Radiology and Radiosurgery, National Centre for Neurosurgery, Astana, Kazakhstan; 5 Hospital Management Sector, National Centre for Neurosurgery, Astana, Kazakhstan; Coventry University, UNITED KINGDOM OF GREAT BRITAIN AND NORTHERN IRELAND

## Abstract

**Background:**

Chronic carotid artery occlusive disease is associated with a high risk of ischemic events and long-term neurological deficits. Optimising surgical revascularisation outcomes requires an integrated understanding of anatomical, hemodynamic, and functional factors. This study aims to assess the neuro-radiological, clinical, and cognitive outcomes following cerebral revascularisation in patients with chronic internal carotid artery occlusion.

**Methods:**

This single-centre, prospective, open-label interventional longitudinal study includes 60 patients with chronic internal carotid artery occlusion undergoing superficial temporal artery-to-middle cerebral artery (STA–MCA) bypass at National Centre for Neurosurgery in Kazakhstan. The primary endpoint of the study is the incidence of ischemic stroke during the 6-month follow-up period after surgery. Pre- and postoperative evaluations include detailed angiographic assessment of collateral circulation and donor vessel diameter, as well as cerebral perfusion imaging using arterial spin labelling MRI. Neurological and cognitive function are assessed using standardized clinical and neuropsychological tests. Early and six-month follow-up evaluations focus on bypass patency, perfusion changes, and clinical outcomes.

**Discussion:**

By integrating anatomical, hemodynamic, and functional data, this study aims to identify predictors of successful revascularisation and favorable clinical outcomes. Correlation of postoperative angiographic and perfusion findings with cognitive and neurological recovery may refine surgical decision-making, enhance risk stratification, and optimize individualized patient care. Ultimately, this comprehensive approach seeks to improve the overall efficacy and safety of STA-MCA bypass in patients with chronic carotid artery occlusive disease.

**Trial registration:**

ClinicalTrials.gov NCT07396025. Registered on February 5, 2026.

## Introduction

Cerebrovascular diseases remain a major global health challenge because of their substantial clinical, societal, and economic burden. According to the Global Burden of Disease 2021 study, stroke is the second leading cause of mortality among non-communicable diseases worldwide and the third leading cause of death and disability combined, accounting for nearly 7 million deaths annually In 2021, approximately 93.8 million individuals were living with the consequences of stroke, while 11.9 million new stroke events were reported globally [1]. Ischemic stroke accounted for approximately 65.3% of all stroke cases [2], with large vessel occlusion implicated in up to 46% of ischemic events [3].

A major challenge in chronic carotid artery occlusive disease is identifying patients most likely to benefit from surgical revascularization while optimizing treatment strategies and understanding postoperative neurological and cognitive outcomes. The role of extracranial–intracranial (EC -IC) bypass remains controversial. The Carotid Occlusion Surgery Study (COSS) reported no reduction in recurrent ischemic stroke despite improved cerebral hemodynamics following bypass [4,5]. Conversely, the Japanese EC -IC Bypass Trial (JET) and JET-2 study suggested potential benefits of revascularization in carefully selected patients with documented hemodynamic compromise [6,7]. Subsequent observational studies have reported similar findings in selected high-risk populations [8,9]. These inconsistent results highlight the need for further investigation into postoperative hemodynamic, neurological, and cognitive outcomes to improve patient selection and surgical decision-making.

Key factors influencing long-term outcomes after direct EC-IC bypass include graft patency, stroke incidence, and functional recovery [10]. These outcomes are determined by the precision of the surgical technique, baseline and postoperative hemodynamic characteristics, and the extent of cerebral revascularisation achieved. However, there is a limited amount of high-quality longitudinal evidence evaluating the relationship between postoperative collateral flow development and long-term neurological and cognitive recovery. In particular, whether improvements in cerebral hemodynamics translate into meaningful functional and neurocognitive benefits remains insufficiently characterized. This knowledge gap limits the ability to optimize patient selection and refine prognostic assessment after revascularization, providing an important rationale for the present study.

Although emergency EC-IC bypass for acute strokes has demonstrated positive neurological outcomes [11,12], its effect on long-term neurological recovery in patients with chronic steno-occlusive cerebrovascular disease has not been sufficiently investigated. In patients with chronic internal carotid artery (ICA) occlusion and documented cerebral hypoperfusion, the long-term neurological and cognitive effects of superficial temporal artery–to–middle cerebral artery (STA-MCA) bypass remain insufficiently characterized. Existing studies evaluating postoperative cognitive outcomes after EC-IC bypass are limited by small sample sizes, heterogeneous methodologies, and incomplete longitudinal follow-up [13–15]. Furthermore, the relationship between postoperative cerebral perfusion improvement and neurological or cognitive recovery has not been systematically investigated. This evidence gap limits understanding of the functional consequences of revascularization and highlights the need for prospective studies evaluating hemodynamic, neurological, and cognitive outcomes following STA-MCA bypass in this high-risk population.

To address these knowledge gaps, this prospective single-arm interventional cohort study aims to evaluate hemodynamic, neurological, and cognitive outcomes following STA-MCA bypass in patients with chronic ICA occlusion and documented cerebral hypoperfusion. The primary endpoint is the incidence of recurrent ischemic cerebrovascular events, defined as ischemic stroke or transient ischemic attack occurring within 6 months after surgery. The secondary endpoints include postoperative neurological and cognitive improvement, assessed using standardized neurological and neuropsychological measures, and their relationship with changes in cerebral perfusion assessed using arterial spin labeling magnetic resonance imaging and angiographic parameters of revascularization. Specifically, the study aims to investigate whether postoperative hemodynamic improvement correlates with neurological recovery and cognitive trajectories following revascularization. By integrating radiological, perfusional, and functional assessments, this study seeks to improve prognostic evaluation and refine patient selection for surgical revascularization in chronic carotid artery occlusive disease.

### Study objectives

#### Primary objective.

To evaluate the incidence of ischemic stroke during the 6-month follow-up period after surgery.

#### Secondary objective.

To assess early postoperative haemodynamic changes, graft patency, changes in neurological and cognitive outcomes at 6 months, all-cause mortality at 6 months, perioperative complications, and the extent of cerebral revascularisation on follow-up imaging.

## Methods and analysis

### Study design

**Fig 1:**
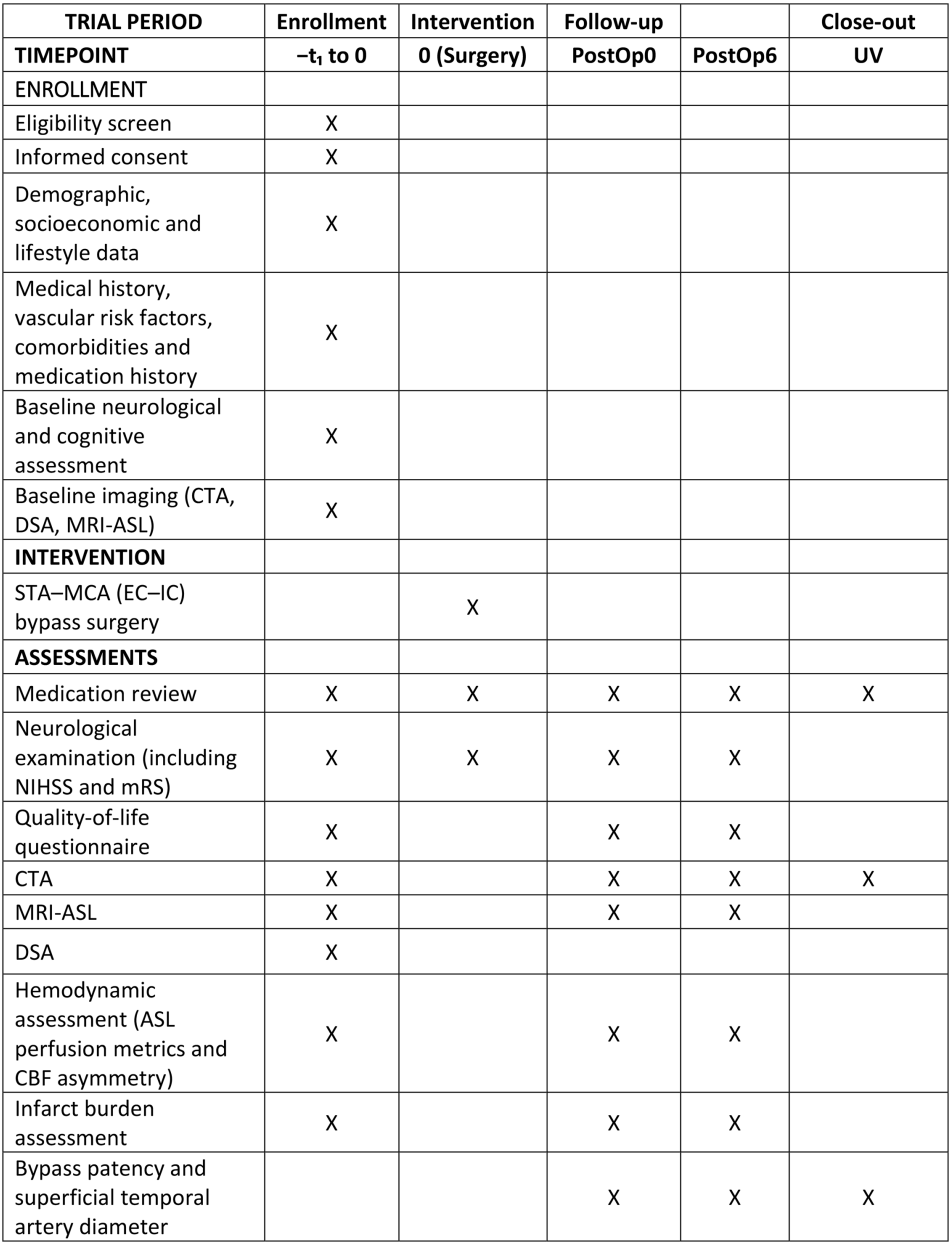
Participant timeline: Schedule of enrollment, interventions, and assessments. Abbreviations: Preop – preoperatively, PostOp0 – Immediately postoperatively, PostOp6–6 months postoperatively, UV – Unscheduled Visit.

This study is a prospective, single-arm, single-centre, interventional cohort study according to the 2025 SPIRIT (Standard Protocol Items: Recommendations for Interventional Trials) Statement (S1 Appendix) and SPIRIT (Fig 1).

### Study population, settings and eligibility criteria

#### Study Population.

Patients with chronic ICA occlusion who underwent elective STA-MCA bypass surgery were enrolled between July 2025 and December 2027.

#### Study setting.

This single-centre study was conducted at the Department of Vascular and Functional Neurosurgery of the National Centre for Neurosurgery, a tertiary referral institution located in the capital city that provides neurosurgical care to the entire national population. With a capacity of 160 beds, the Centre represents the only specialized neurosurgical facility of its kind in Kazakhstan and serves as a regional referral hub for neighboring Central Asian countries [16].

#### Patient selection.

The study requires a multidisciplinary team comprising a neurologist, neurosurgeon, neuropsychologist, neuroradiologist, biostatistician, and research administrator.

Patients will be identified through the Department of Vascular and Functional Neurosurgery. STA-MCA bypass procedures for patients with chronic ICA occlusion will be performed by a team of neurosurgeons within the department. Neurosurgeons must have experience in vascular anastomosis and have successfully conducted STA-MCA bypass surgery on at least 10 patients. Neurological and cognitive assessments will be conducted by a consultant neurologist and neuropsychologist. All radiological imaging will be independently reviewed and interpreted by two radiologists from the Department of Radiology.

Baseline symptomatic status will be incorporated as an adjustment variable in multivariable and longitudinal analyses to account for differences in baseline risk and recovery potential. Exploratory subgroup analyses by symptom status will be performed where sample size permits.

### Eligibility criteria

#### Inclusion.

1. Chronic ICA occlusion confirmed by computed tomography angiography (CTA), magnetic resonance angiography (MRA), and digital subtraction angiography (DSA).
2. Evidence of cerebral hypoperfusion on magnetic resonance imaging (MRI) arterial spin labelling (ASL) within the territory of the occluded ICA, defined as a ≥ 30% reduction compared with the contralateral hemisphere.
3. Modified Rankin Scale (mRS) score of 0–3 at baseline.
4. Preserved language comprehension, with no more than mild motor aphasia and no significant cognitive impairment.
5. Adults aged 18–80 years.
6. Provision of written informed consent by the patient or a legally authorised representative.
7. Willingness and ability to comply with follow-up assessments.

#### Exclusion.

1. Large territorial infarction or marked cerebral atrophy on MRI.
2. Bilateral ICA occlusion or concomitant neurovascular disease (e.g., cerebral aneurysm or arteriovenous malformation).
3. Severe systemic comorbidities, life expectancy <2 years, or a mRS score >3.
4. Cardiac conditions that are associated with a high risk of cerebral embolism (e.g., prosthetic heart valves, infective endocarditis, intracardiac thrombus, sick sinus syndrome, cardiac myxoma, or cardiomyopathy with left ventricular ejection fraction <25%; atrial fibrillation, patent foramen ovale, and atrial septal aneurysm are not exclusion criteria).
5. Contraindications to MRI, CTA, DSA, iodinated contrast agents, or required perioperative antiplatelet therapy.
6. Non-functioning STA-MCA bypass.
7. Participation in another interventional or experimental study within the previous 12 months, including those involving ionizing radiation.
8. Pregnancy.

## Study procedure and intervention

### Clinical and radiological assessments

#### Radiological assessment.

The assessment schedule is given in Table 1. To evaluate the ICA occlusion, a three-level assessment will be conducted using CTA of the head and neck vessels, MRI ASL and Time-of-flight (TOF) sequences, and a confirmatory assessment of occlusion during DSA. All imaging studies will be performed and interpreted by neuroradiologists and neurosurgeons, with findings reviewed collaboratively.

**Table 1:** Data collection schedule.

Assessments	Preop	PostOp0	PostOp6	UV
**Demographic and socioeconomic data, lifestyle factors**				
Age, sex, place of birth, marital status, nationality, employment status, occupation, level of education, smoking status (past, current, never), and alcohol consumption (none, occasional, moderate, and heavy consumption), dietary habits, physical activity, sleep quality, and vacation frequency	+			
**Medical data**				
Timing and characteristics of prior TIA or stroke	+			
Vascular risk factors (hypertension, diabetes mellitus, dyslipidemia, atrial fibrillation	+			
Comorbid conditions (including genetic disorders), history of seizures	+			
Past and current medication use	+	+	+	+
Body mass index (BMI)	+			
**Neurological and cognitive**				
Baseline neurological examination findings	+			
Modified Rankin Scale (mRS)	+	+	+	+
National Institutes of Health Stroke Scale (NIHSS) scores	+	+	+	
Quality-of-life measures (questionnaire)	+	+	+	
Cognitive reserve indicators (educational attainment and occupational complexity), and engagement in cognitively stimulating activities	+			
**Imaging examinations**				
CTA	+	+	+	+
DSA	+			
MRI ASL	+	+	+	
**Hemodynamic parameters**				
Collateral circulation patterns	+			
Location and length of the internal carotid artery occlusion	+			
Circle of Willis configuration	+			
ASL perfusion metrics	+	+	+	
Cerebral blood flow (CBF) asymmetrical indices	+	+	+	
Infarct burden	+	+	+	
White matter hyperintensities	+			
Bypass patency and the diameter of STA		+	+	+

Abbreviations: Preop – preoperatively, PostOp0 – Immediately postoperatively, PostOp6–6 months postoperatively, UV – Unscheduled Visit, TIA – transient ischemic attack, BMI – Body mass index.

Included patients will undergo CTA of the head and neck vessels with a slice thickness of 0.5-0.625 mm to confirm ICA occlusion and to measure the diameter of the donor STA branch at 2 mm distal to its bifurcation.

MRI perfusion studies will be conducted before surgery on 1.5 or 3 Tesla MRI to evaluate cerebral blood flow (CBF) and determine the extent of cerebrovascular insufficiency. The main perfusion method will be ASL, which will provide quantitative CBF values, assess the degree of perfusion deficit, and reveal signs of compensatory collateral circulation based on the presence of arterial transit artifacts (ATA). Imaging parameters are described in in S1 Table.

DSA will be performed using a biplane digital subtraction angiography system (Artis Zee, Siemens) with a 6F guiding catheter at a frame rate of 7.5 frames per second. The angiographic assessment will evaluate the presence of communicating arteries, the completeness of the Circle of Willis, and the extent of collateral circulation. Hemodynamic compensation will be quantified by analyzing filling times and phase delays across the arterial, parenchymal, and venous phases, expressed in frames. Image analysis will be conducted using Siemens Artis Q angiography software to ensure accurate and reproducible measurements.

#### Neurological assessment.

The degree of neurological deficit before and after surgery will be evaluated by neurologist using the Baseline neurological examination findings, mRS and National Institutes of Health Stroke Scale (NIHSS) scores. The consequences of prior stroke will also be assessed with the mRS [15] and the Functional Activities Questionnaire (FAQ) [17]. Preoperatively and six months after surgery, all patients will be evaluated under a unified diagnostic protocol, with results collected and recorded separately before entering them into a general database with strict personal data encoding.

#### Cognitive function assessment.

Changes in cognitive function before and after surgery and six months postoperatively will be assessed using the neuropsychological tests, measuring both verbal and non-verbal IQ.

A standardized battery of neuropsychological tests will be administered by a psychologist to evaluate global cognition, memory, visuospatial skills, and executive function (S2 Table). The Montreal Cognitive Assessment (MoCA) will be used as a screening tool for global cognitive function, assessing domains including attention, executive functions, memory, language, visuospatial skills, abstraction, and orientation [18]. The Rey Auditory Verbal Learning Test (RAVLT) will be employed to evaluate verbal learning and memory performance, including immediate recall, delayed recall, and recognition memory [19]. Nonverbal test of Kohs Block Design will be used to assess visuospatial reasoning, problem-solving, and perceptual-motor coordination by requiring participants to reproduce geometric patterns with colored blocks [20]. The Stroop Test will be applied to measure selective attention, processing speed, and cognitive flexibility by evaluating the ability to inhibit cognitive interference [21].

To minimize potential learning or practice effects associated with repeated testing, assessments will be conducted at predefined intervals with sufficient time between evaluations, and standardized administration procedures will be maintained across all sessions. Whenever available, alternative test versions will be used for repeated assessments.

Neuropsychological test performance will be interpreted using age- and education-adjusted normative data when available, in order to reduce the potential confounding effect of educational background on cognitive outcomes.

#### Perioperative management.

Perioperative management was standardized for all patients to minimize treatment variability and potential confounding effects on outcomes. General anesthesia was performed using a standardized protocol with continuous hemodynamic monitoring. Intraoperative blood pressure was strictly controlled to avoid hypotension or excessive fluctuations that could compromise cerebral perfusion or graft flow. Postoperatively, patients were monitored in a dedicated intensive care unit with close neurological interventional and continuous blood pressure control. Systolic blood pressure targets were maintained within a predefined range to ensure adequate bypass perfusion while minimizing the risk of hyperperfusion-related complications.

All patients received standardized antiplatelet therapy with aspirin 100 mg once daily, initiated preoperatively or immediately postoperatively according to the institutional protocol, and continued throughout the follow-up period unless contraindications developed.

#### Surgical treatment.

Eligible patients with chronic ICA occlusion will undergo standard STA-MCA bypass surgery. The procedure will be carried out using a standardized microsurgical end-to-side anastomosis technique. Intraoperative selection of the donor STA branch and recipient MCA segment will be based on vessel diameter, flow characteristics, and surgical accessibility. Intraoperative bypass patency will be assessed using the “faucet” technique” [22] in combination with microscopic indocyanine green (ICG) angiography to ensure adequate flow through the anastomosis.

#### Intraoperative perfusion and blood flow direction assessment.

Intraoperative perfusion and blood flow direction will be assessed using ICG fluorescent angiography with the Carl Zeiss Pentero 800 microscope equipped with FLOW 800 technology, which allows real-time visualization of cerebral vessels. All data will be processed and analyzed to create a color map depicting hemodynamic and perfusion delays. This enables comparison of perfusion parameters in the anastomosis region before and after bypass surgery, as well as assessment of blood flow direction and hyperperfusion zones in the brain. The assessment will be performed by neurosurgeons.

#### Postoperative study procedures.

Within 1-3 days after surgery, CTA of the head and neck will be performed to confirm bypass patency, measure the caliber of the donor STA branch, and visualize its course. At 3–5 days postoperatively, ASL-based MR perfusion imaging will be conducted to evaluate cerebral perfusion changes compared with preoperative studies. 3D segmentation and quantitative perfusion mapping will be applied to provide an objective assessment of regional perfusion improvements following surgery. Before hospital discharge, all patients will undergo a repeat evaluation of neurological status and cognitive function using the same standardised tests as preoperatively.

#### Follow-up assessment.

At six months after surgery, all patients will undergo follow-up assessment including CTA of the head and neck to evaluate bypass patency and measure the caliber of the donor STA branch, MR perfusion imaging with 3D segmentation to assess cerebral perfusion changes, and repeat neurological and neuropsychological evaluations using the same tests administered preoperatively and at discharge (NIHSS, mRS, MoCA, RAVLT, Kohs Block Design, and Stroop tests). The planning of the follow-up is described in Table 1.

### Study endpoints

#### Primary endpoint.

To evaluate the incidence of ischemic stroke during the 6-month follow-up period after surgery.

#### Secondary endpoints.

Early postoperative graft patency, perioperative safety, neurological and cognitive outcomes, recurrent cerebrovascular events, mortality, and radiological markers of revascularisation.

#### Definitions of outcomes.

The primary outcome is defined as the incidence of ischemic stroke during the 6-month follow-up period after surgery. This outcome will be assessed based on clinical evaluation and confirmed by neuroimaging (MRI or CT) in cases of suspected new neurological deficits, in accordance with standard diagnostic criteria for ischemic stroke.

Secondary outcomes included early postoperative haemodynamic change (≤7 days) assessed using the same imaging modality; graft patency was defined as uninterrupted flow through the bypass on intraoperative ICG and postoperative CTA; changes in cerebral haemodynamic status from baseline to 6 months after surgery will be assessed using ASL MRI and defined as a quantitative increase in regional CBF in the affected hemisphere and/or a reduction in interhemispheric perfusion asymmetry compared with baseline; change in neurological status from baseline to 6 months assessed using the NIHSS and the mRS; changes in cognitive function from baseline to 6 months based on standardised neuropsychological testing; all-cause mortality at 6 months; perioperative complications within 30 days (including intracranial haemorrhage, hyperperfusion syndrome, seizures, wound complications, and graft occlusion); and angiographic extent of revascularisation on follow-up imaging.

### Data management

The collected data will be meticulously entered into a database, where revascularisation progress, the development of collateral circulation, and perfusion changes correlated with neurological and cognitive shifts will be evaluated. All data will undergo statistical processing and analysis to identify patterns and correlations, as well as survival analysis to assess collateral development and psychoneurological dynamics. All participants are expected to complete study visits over the planned 6-month follow-up period.

#### Sample size calculation.

Sample size estimation was performed in Stata (version 19.5) using the power pairedmeans command, based on a paired pre-post comparison of the primary outcome. Assuming a two-sided α of 0.05, 80% power, a standardised effect size of 0.40, and a within-subject correlation of 0.5, 52 evaluable patients were required. Allowing for potential loss to follow-up, a target sample size of 60 patients was set, providing approximately 86% power. Sensitivity analyses indicated that power ranged from 73% to 98% across plausible within-subject correlations (0.3–0.7).

### Statistical analysis

Continuous variables will be reported as mean with standard deviation or median with interquartile range, depending on distribution. Categorical variables will be presented as frequencies and percentages. The primary outcome, change in cerebral hemodynamic status from baseline to 6 months, will be analyzed using a paired t-test or Wilcoxon signed-rank test, depending on data distribution. Mean change in CBF and/or perfusion asymmetry will be reported with 95% confidence intervals.

Early postoperative and 6-month changes in neurological and cognitive scores will be analyzed using paired tests. Categorical outcomes, including graft patency, recurrent TIA or stroke, complications, and mortality, will be reported as proportions with 95% confidence intervals.

Where appropriate, exploratory regression analyses will be performed to identify factors associated with hemodynamic improvement and clinical outcomes. Potential predictors may include age, sex, baseline perfusion deficit, vascular risk factors, duration of symptoms, and preoperative neurological status. Given the limited sample size, multivariable models will be restricted to a small number of clinically relevant covariates.

Because repeated measurements will be obtained from the same participants, analyses will account for within-subject correlation. Continuous repeated outcomes will be analysed using linear mixed-effects models, with time point entered as a fixed effect and participant ID as a random intercept. This approach allows estimation of postoperative change while accounting for correlation between repeated measures within individuals and accommodating incomplete follow-up data under a missing-at-random assumption.

A p-value <0.05 will be considered statistically significant. All analysis will be performed using Stata 19.5.

#### Mortality analysis.

All-cause mortality will be assessed 6 months after surgery. Mortality will be reported as the number and proportion of deaths with exact 95% confidence intervals.

Time-to-event analysis will be performed using Kaplan-Meier survival curves, with survival probability reported for the follow-up period. Patients alive at the last follow-up will be censored on the date of last clinical contact. Considering the cohort of 60 patients, Cox regression will not be performed unless the number of events is sufficient.

#### Missing data handling.

Missing data will be evaluated for extent and pattern. Longitudinal analyses will use mixed-effects models, which account for within-subject correlation and allow inclusion of incomplete observations under a missing-at-random assumption. Where appropriate, multiple imputations by chained equations will be used, with sensitivity analyses performed to assess robustness. For time-to-event outcomes, patients will be censored at the last follow-up.

### Ethics

Prior to registration, all participants will provide written informed permission in compliance of the Declaration of Helsinki’s ethical guidelines.

Patient confidentiality will be ensured through strict coding and anonymization of personal identifiers and medical record identification numbers (MRINs).

Institutional Review Board Statement: The study will adhere strictly to scientific ethics principles, including prevention of data fabrication, falsification, plagiarism, and false authorship. The study protocol and other necessary documents were reviewed and approved by the ethics committee (Protocol No. 4 of September 25, 2024, S2 Appendix). This study was retrospectively registered on ClinicalTrials.gov (NCT07396025; February 5, 2026) due to an administrative delay that prevented registration before participant enrolment; however, the study protocol, eligibility criteria, intervention, and outcome measures were predefined prior to recruitment and remained unchanged after registration. This study will be conducted according to the principles of the Declaration of Helsinki and subsequent amendments.

Informed Consent Statement: Written informed consent has been obtained from the patient(s) to publish this paper.

The authors confirm that all ongoing and related trials for this drug/intervention are registered.

## Discussion

This prospective single-arm interventional cohort study is designed to evaluate hemodynamic, neurological, and cognitive outcomes following STA-MCA bypass in patients with chronic ICA occlusion and documented cerebral hypoperfusion. Despite advances in evidence-based medical management, recurrent ischemic events remain common in selected patients with large-vessel cerebrovascular disease, particularly among those with persistent hemodynamic compromise [22,23]. Although EC–IC bypass remains controversial, accumulating evidence suggests that surgical revascularization may still represent a therapeutic option for carefully selected high-risk patients with recurrent ischemic symptoms despite optimized medical therapy [24,25].

A major challenge in chronic carotid artery occlusive disease is understanding the relationship between postoperative hemodynamic improvement and functional recovery. Previous studies of EC-IC bypass have largely focused on recurrent stroke prevention, graft patency, and perioperative safety, while substantially less attention has been directed toward postoperative neurological and cognitive trajectories [25]. This gap in evidence is particularly important because restoration of CBF may influence not only recurrent ischemic risk but also neurological recovery and cognitive function, outcomes that remain insufficiently characterized in patients with chronic ICA occlusion.

To address this limitation, the present study incorporates a multidimensional postoperative assessment integrating neuroradiological, hemodynamic, neurological, and neuropsychological measures. Specifically, repeated ASL MRI will be used to quantify cerebral perfusion changes after surgery, while angiographic assessment will characterize collateral circulation and donor vessel adaptation. These measures will be correlated with longitudinal changes in neurological and cognitive function to determine whether postoperative hemodynamic improvement translates into clinically meaningful recovery.

The use of ASL perfusion imaging represents an important strength of this protocol. Unlike contrast-enhanced techniques, ASL permits quantitative, non-invasive assessment of CBF without exogenous tracers, facilitating repeated longitudinal measurements after revascularization [26–28]. Although ASL has demonstrated value in predicting postoperative hemodynamic improvement after revascularization in conditions such as moyamoya disease, evidence in chronic ICA occlusion remains limited [26,27]. By incorporating serial perfusion imaging, the present study may improve understanding of the temporal relationship between cerebral perfusion restoration and postoperative recovery.

Existing studies investigating cognitive outcomes following EC–IC bypass remain limited by small sample sizes, heterogeneous methodologies, and incomplete follow-up. The Randomized Evaluation of Carotid Occlusion and Neurocognition (RECON) study, an ancillary investigation of the COSS trial, found no significant differences in cognitive outcomes between surgical and medical treatment groups; however, interpretation was limited by premature trial termination and a small sample size [29]. Similar limitations were reported in another prospective study, in which only a small proportion of screened patients completed longitudinal neuropsychological assessment [30]. More recently, Yu Duan et al. demonstrated that postoperative cerebral perfusion improvement following EC-IC bypass in elderly patients with chronic middle cerebral artery occlusion was associated with improved cognitive function [32]. Nevertheless, the study was restricted to middle cerebral artery disease and primarily relied on the MoCA, which may not adequately capture domain-specific cognitive changes following revascularization.

In our study the MoCA will be used as a global cognitive screening instrument because of its established sensitivity for detecting mild cognitive impairment and executive dysfunction, particularly in patients with vascular cognitive impairment [18]. However, as a screening tool, the MoCA alone may insufficiently characterize subtle domain-specific postoperative cognitive changes, particularly single-domain impairments [23]. Therefore, additional neuropsychological tests were incorporated to assess cognitive domains potentially affected by chronic cerebral hypoperfusion and altered cerebral hemodynamics.

The RAVLT was selected to assess verbal learning and memory processes, including immediate recall, delayed recall, and recognition memory, which may be sensitive to functional changes in cerebral perfusion and network connectivity [24]. Previous studies have demonstrated the utility of the RAVLT in detecting subtle memory dysfunction and in functional neuroimaging paradigms evaluating verbal memory processing [25]. In addition, the test has been validated across different populations and age groups, supporting its applicability in longitudinal cognitive assessment [31].

The Kohs Block Design Test was included to assess visuospatial abilities, perceptual organization, and nonverbal executive functioning, domains commonly affected in cerebrovascular disease and widely evaluated through constructional praxis and block design paradigms in neuropsychological assessment [26]. The test reflects visuospatial constructional ability and nonverbal problem-solving skills, which are important determinants of functional independence in activities of daily living, including post-stroke recovery [27].

The Stroop Color and Word Test was used to assess executive function, particularly inhibitory control and selective attention, which are commonly impaired after stroke. Its proven reliability, validity, and responsiveness in stroke populations further support its suitability for detecting and monitoring cognitive dysfunction in this setting [21,28,29].

Intraoperative assessment of cerebral hemodynamics represents another understudied aspect of revascularization surgery. ICG fluorescence angiography has demonstrated utility in assessing intraoperative perfusion and predicting complications such as cerebral hyperperfusion syndrome [30]. However, the relationship between postoperative graft adaptation, changes in STA caliber, cerebral perfusion improvement, and subsequent neurological or cognitive recovery has not been systematically investigated. The present study seeks to address this gap through combined angiographic and perfusion-based assessment.

Importantly, this study is not designed to evaluate the comparative efficacy or effectiveness of EC-IC bypass relative to conservative treatment, as no control group is included. Rather, the objective is to characterize recurrent cerebrovascular events, postoperative hemodynamic changes, and neurological and cognitive trajectories following STA-MCA bypass in a carefully selected cohort of patients with chronic ICA occlusion and documented cerebral hypoperfusion. This approach is intended to generate clinically meaningful evidence regarding recovery patterns and prognostic indicators that may inform future comparative studies and improve patient selection for surgical revascularization.

Overall, by integrating recurrent ischemic outcomes with perfusional, neurological, and neurocognitive assessments, the present study aims to improve understanding of the functional consequences of cerebral revascularization in chronic carotid artery occlusive disease. Better characterization of the relationship between cerebral perfusion restoration and clinical recovery may support more individualized surgical decision-making and improve prognostic assessment in patients undergoing STA-MCA bypass.

### Limitations

This study has several limitations. First, its single-center, open-label design may limit the generalizability of the findings and introduces a potential risk of selection bias. However, conducting the study in a high-volume tertiary neurosurgical center ensures consistency in surgical technique, perioperative management, and imaging protocols.

Second, the absence of a non-surgical control group precludes direct comparison between surgical revascularisation and best medical therapy. Nevertheless, the primary objective of this study is not to reassess the efficacy of STA-MCA bypass versus conservative treatment, but rather to investigate detailed hemodynamic, neurological, and cognitive changes following bypass surgery in a carefully selected population with documented cerebral hypoperfusion.

Third, the planned sample size of 60 participants was primarily determined based on feasibility considerations, including the recruitment capacity of our center, the predefined study duration, and the scope of the funded grant project. As a result, the planned sample size may limit statistical power for detecting small effect sizes and for building robust predictive models. Therefore, multivariable analyses will be considered exploratory, and results will be interpreted cautiously. Larger multicenter studies will be required to externally validate the findings.

Fourth, the single-arm study design without a control group limits the ability to control for confounding factors and precludes definitive causal inference regarding the observed effects. As a result, the findings should be interpreted as associative, and residual confounding cannot be excluded despite adjustment in exploratory analyses.

Fifth, cognitive assessment may be influenced by learning effects, mood, fatigue, or educational background. To mitigate these factors, standardized testing conditions will be applied, parallel test versions will be used when available, and cognitive reserve indicators will be incorporated into the analysis.

Sixth, advanced imaging techniques such as ASL MRI and intraoperative ICG FLOW800 analysis require specialized equipment and expertise, which may limit reproducibility in lower-resource settings. However, these techniques provide unique insights into cerebral hemodynamics and represent a major strength of the study.

Finally, the follow-up period of six months may not capture long-term neurological and cognitive trajectories or delayed graft failure. Extended follow-up is planned in future studies to assess durability of revascularisation and long-term clinical outcomes.

## Supporting information

S1 Table Parameters of sequences on 3.0-T General Electric Signa Architect. (DOCX)

S2 Table Tests for assessment of cognitive function. (DOCX)

S1 Appendix SPIRIT 2025 checklist of items to address in a randomized trial protocol. (PDF)

S2 Appendix Bioethics protocol. (DOCX)
