# Supplementary material for: Hemodynamic, neurological, and cognitive outcomes after surgical revascularisation in occlusive cerebrovascular disease: Study protocol for single-arm interventional cohort study
Source: PLoS One. 2026 Aug 7;21(8):e0354103. doi: 10.1371/journal.pone.0354103 (PMC13450772; doi:10.1371/journal.pone.0354103)
Supplement: Supplementary file 2 [file pone.0354103.s001.docx]

S1 Table. Parameterss of sequences on 3.0-T General Electric Signa Architect (TOF - time of flight; T1 - T1-weighted imaging; T2 - T2-weighted imaging; ASL - arterial spin labeling; TR - repetition time; TE - echo time; FOV - field of view; FLAIR - Fluid-Attenuated Inversion Recovery; AX – axial).

| **Parameters** | **AX 3D-TOF SPGR** | **AxT2-PROPELLER** | **3D- Ax T2FLAIR** | **3D- ASL** |
| --- | --- | --- | --- | --- |
| **TR(ms)** | 24,0 | 6427 | 6000 | 4795,0 |
| **TE(ms)** | 3.74 | 100.0 | 105.0 | 10,7 |
| **FOV(mm^2^)** | 24,0 | 22.0 | 22.0 | 24.0 |
| **Matrix** | 320x192 | 360 | 224x224 | 512 |
| **Slice thickness(mm)** | 1,00 | 3.0 | 0.70 | 4 |
| **Echo train(ms)** | - | 32 | 220 | - |
| **Bandwidth(Hz/pixel)** | 62,50 | 50.00 | 35,71 | 62,50 |
| **Scan time** | 3 min 40s | 93min 01s | 7 min 27s | 4 min 27s |
