# Supplementary material for: Hemodynamic, neurological, and cognitive outcomes after surgical revascularisation in occlusive cerebrovascular disease: Study protocol for single-arm interventional cohort study
Source: PLoS One. 2026 Aug 7;21(8):e0354103. doi: 10.1371/journal.pone.0354103 (PMC13450772; doi:10.1371/journal.pone.0354103)
Supplement: Supplementary file 3 [file pone.0354103.s002.docx]

**S2 Table.** Tests for Assessment of Cognitive Function.

| **Test** | **Brief Description** | **Advantage** |
| --- | --- | --- |
| **MoCA (Montreal Cognitive Assessment)** | A brief screening tool for mild cognitive impairment, assessing multiple cognitive domains | Quick and comprehensive; sensitive for early cognitive decline |
| **RAVLT (Rey Auditory Verbal Learning Test)** | Assesses verbal learning and memory through repeated recall of a word list | Measures different memory processes (learning, recall, recognition) |
| **Kohs Block Design Test** | A nonverbal test where participants replicate geometric patterns using colored blocks | Useful for evaluating visuospatial skills, less affected by language. |
| **Stroop Test** | Measures attention, processing speed, and cognitive flexibility by naming ink colors of printed words | Sensitive to executive function and inhibitory control impairments |
