# Supplementary material for: Hemodynamic, neurological, and cognitive outcomes after surgical revascularisation in occlusive cerebrovascular disease: Study protocol for single-arm interventional cohort study
Source: PLoS One. 2026 Aug 7;21(8):e0354103. doi: 10.1371/journal.pone.0354103 (PMC13450772; doi:10.1371/journal.pone.0354103)
Supplement: Supplementary file 4 [file pone.0354103.s004.docx]

JSC "NATIONAL CENTER FOR NEUROSURGERY"

**RESEARCH PROTOCOL**

**«**Hemodynamic, Neurological, and Cognitive Outcomes after Surgical Revascularization in Occlusive Cerebrovascular Disease: Study Protocol for a Prospective Single-Center Trial**»**

**Principle Investigator:** Asylbek Kaliev, neurosurgeon, PhD

**Main Executor:** Chingiz Nurimanov, neurosurgeon, PhD

**Location:** JSC "National Center for Neurosurgery", Turan Avenue 34/1, 010000, Astana

**I Purpose of the study:** Improving the effectiveness of surgical treatment for patients with stenotic-occlusive cerebrovascular diseases through a comprehensive analysis of the clinical picture, perfusion studies, intraoperative flowmetry, and angiographic data, which facilitates timely identification of indications for surgical interventions and minimizes the risk of complications.

**II Introduction**

Acute cerebrovascular accidents remain one of the leading causes of disability and mortality among older patients. A key cause of ischemic strokes is atherosclerotic involvement of the brachiocephalic arteries, including carotid artery occlusion.

This study aims to improve the surgical treatment of patients with stenotic-occlusive cerebrovascular disease through a comprehensive analysis of neuroradiological data, perfusion parameters, and the clinical picture. Special attention is given to assessing the degree of cerebral revascularization after surgery using angiography-based quantitative evaluation (EIKMA). This approach allows for more precise determination of indications for surgical intervention, improves predictions of functional outcomes, and minimizes the risk of postoperative complications, ultimately enhancing the overall effectiveness of treatment for this patient population.

**III. Inclusion criteria**

**1.Number of participants:** The study plans to include 60 patients.

**2. Study site:** Department of Vascular and Functional Neurosurgery

**3. Sex distribution:** No restrictions.

**4. Age range:** 18 to 70 years.

**5. Ethnicity:** No ethnic restrictions.

**6. Exclusion criteria:**

1**.** Patients with large territorial infarction or marked cerebral atrophy on MRI.

2. No evidence of regional hypoperfusion on ASL MRI relative to the contralateral hemisphere

3. Patients with a non-functioning STA-MCA bypass

4. Patients aged under 18

5. Severe systemic comorbidities, life expectancy <2 years, or a modified Rankin Scale (mRS) score >3.

6. Patients with bilateral ICA occlusion, neurovascular disease (cerebral aneurysm or arteriovenous malformation)

7. Contraindications to MRI, CTA, DSA, iodinated contrast agents, or antiplatelet therapy required in the perioperative period.

8. Cardiac conditions associated with a high risk of cerebral embolism, including prosthetic heart valves, infective endocarditis, intracardiac thrombus, sick sinus syndrome, cardiac myxoma, or cardiomyopathy with left ventricular ejection fraction <25% (atrial fibrillation, patent foramen ovale, and atrial septal aneurysm are not exclusion criteria).

9. Pregnancy.

10. Participation in another interventional or experimental trial within the previous 12 months, including studies involving ionizing radiation].

**Vulnerable groups: None.**

**IV. methods and procedures**

*Patient selection*

Patients with asymptomatic or symptomatic carotid artery occlusion undergoing STA–MCA bypass at the neurosurgical center will be prospectively enrolled between 1^st^ of August 2025 and 31^st^ of December 2027.

Prior to registration, all participants will provide written informed permission in compliance of the Declaration of Helsinki's ethical guidelines. Preoperative evaluation will include computed tomography angiography (CTA), magnetic resonance imaging (MRI), digital subtraction angiography (DSA), and comprehensive neurological and neuropsychological assessments. MRI also will be employed to exclude patients with extensive territorial infarctions. Only patients with radiologically confirmed ICA occlusion and arterial spin labeling (ASL) MRI–documented cerebral hypoperfusion in the affected vascular territory who undergo STA–MCA bypass will be included.

The study cohort will be recruited at the National Center for Neurosurgery in Astana through a multidisciplinary collaboration involving neurosurgeons, radiologists, neurologists, and neuropsychologists. All data will be collected using standardized case report forms and subsequently entered into a unified, secure database. Patient confidentiality will be ensured through strict coding and anonymization of personal identifiers and medical record identification numbers (MRINs). Detailed inclusion and exclusion criteria are presented in Table 1.

*Study variables*

Data collection will include the following variables:

1. Demographic characteristics: Age, gender, birthplace, nationality, employment status, profession, and education level.

2. Medical history: Past medical and surgical history, onset of transient ischemic attack (TIA) or stroke, vascular risk factors (hypertension, diabetes mellitus, dyslipidemia, atrial fibrillation), comorbidities (including genetic disorders), previous surgeries, ongoing follow-up care, current medications, and body mass index (BMI).

3. Clinical, neurological, and cognitive status: Baseline neurological examination findings, Modified Rankin Scale (mRS) and National Institutes of Health Stroke Scale (NIHSS) scores, history of seizures, quality-of-life measures, cognitive reserve indicators (educational attainment and occupational complexity), and engagement in cognitively stimulating activities will be assessed.

4. Lifestyle factors: Smoking status, dietary habits, physical activity, alcohol consumption, sleep quality, vacation frequency, tobacco use, and other lifestyle factors.

5. Radiological and hemodynamic parameters: Location and length of the internal carotid artery occlusion; collateral circulation patterns; Circle of Willis configuration; ASL perfusion metrics; cerebral blood flow (CBF) asymmetry indices; infarct burden; white matter hyperintensities; and postoperative bypass patency and the diameter of STA pre and postoperatively.

***Experimental Design***

*Neuroradiological assessment*

To evaluate the ICA occlusion, a three-level assessment will be conducted using instrumental methods. This comprehensive approach includes CTA of the head and neck vessels, specialized MRI ASL and Time-of-flight (TOF) sequences, and a confirmatory assessment of occlusion during DSA. All imaging studies will be performed and interpreted by experienced neuroradiologists and neurosurgeons, with findings reviewed collaboratively.

All patients will undergo CTA of the head and neck vessels with a slice thickness of 0.5–0.625 mm to confirm internal carotid artery occlusion and to measure the diameter of the donor STA branch at 2 mm distal to its bifurcation.

To evaluate CBF and determine the extent of cerebrovascular insufficiency, MRI perfusion studies will be conducted before surgery on 1.5 or 3 Tesla MRI. The main perfusion method will be ASL, which will provide quantitative CBF values, assess the degree of perfusion deficit, and reveal signs of compensatory collateral circulation based on the presence of arterial transit artifacts (ATA). All imaging parameters are described in Appendix A (Table A1).

DSA will be performed using a biplane digital subtraction angiography system (Artis Zee, Siemens) with a 6F guiding catheter at a frame rate of 7.5 frames per second. The angiographic assessment will evaluate the presence of communicating arteries, the completeness of the Circle of Willis, and the extent of collateral circulation. Hemodynamic compensation will be quantified by analyzing filling times and phase delays across the arterial, parenchymal, and venous phases, expressed in frames. Image analysis will be conducted using Siemens Artis Q angiography software to ensure accurate and reproducible measurements.

*Neurological status assessment*

The degree of neurological deficit before and after surgery will be evaluated by neurologist using the Baseline neurological examination findings, Modified Rankin Scale (mRS) and National Institutes of Health Stroke Scale (NIHSS) scores. The consequences of prior stroke will also be assessed with the mRS [15] and the Functional Activities Questionnaire (FAQ) [16]. Preoperatively and six months after surgery, all patients will be evaluated under a unified diagnostic protocol, with results collected and recorded separately before entering them into a general database with strict personal data encoding.

*Cognitive function assessment*

Changes in cognitive function before and after surgery and six months postoperatively will be assessed using the neuropsychological tests, measuring both verbal and non-verbal IQ. A standardized battery of neuropsychological tests will be administered by a psychologist to evaluate global cognition, memory, visuospatial skills, and executive function (Appendix B (Table B1)). The Montreal Cognitive Assessment (MoCA) will be used as a screening tool for global cognitive function, assessing domains including attention, executive functions, memory, language, visuospatial skills, abstraction, and orientation [17]. The Rey Auditory Verbal Learning Test (RAVLT) will be employed to evaluate verbal learning and memory performance, including immediate recall, delayed recall, and recognition memory [18]. Nonverbal test of Kohs Block Design will be used to assess visuospatial reasoning, problem-solving, and perceptual-motor coordination by requiring participants to reproduce geometric patterns with colored blocks [19]. The Stroop Test will be applied to measure selective attention, processing speed, and cognitive flexibility by evaluating the ability to inhibit cognitive interference [20].

*Surgical treatment*

All eligible patients with ICA occlusion will undergo standard STA–MCA bypass surgery performed by experienced neurosurgeons. The procedure will be carried out using a standardized microsurgical end-to-side anastomosis technique, which is well established and routinely performed by the participating surgeons [21]. Intraoperative selection of the donor STA branch and recipient MCA segment will be based on vessel diameter, flow characteristics, and surgical accessibility. Intraoperative bypass patency will be assessed using the “faucet" technique” [21] in combination with microscopic indocyanine green (ICG) angiography to ensure adequate flow through the anastomosis.

*Intraoperative perfusion and blood flow direction assessment*

Intraoperative perfusion and blood flow direction will be assessed using indocyanine green fluorescent angiography with the Karl Zeiss Pentera 800 microscope equipped with FLOW 800 technology, which allows real-time visualization of cerebral vessels. All data will be processed and analyzed to create a color map depicting hemodynamic and perfusion delays. This enables comparison of perfusion parameters in the anastomosis region before and after bypass surgery, as well as assessment of blood flow direction and hyperperfusion zones in the brain. The assessment will be performed by neurosurgeons.

*Postoperative study procedures*

Within 1–3 days after surgery, CTA of the head and neck will be performed to confirm bypass patency, measure the caliber of the donor STA branch, and visualize its course. At 3–5 days postoperatively, ASL-based MR perfusion imaging will be conducted to evaluate cerebral perfusion changes compared with preoperative studies. 3D segmentation and quantitative perfusion mapping will be applied to provide objective assessment of regional perfusion improvements following surgery. Prior to hospital discharge, all patients will undergo a repeat evaluation of neurological status and cognitive function using the same standardized tests as preoperatively.

*Follow-up assessment*

At six months after surgery, all patients will undergo follow-up assessment including CTA of the head and neck to evaluate bypass patency and measure the caliber of the donor STA branch, MR perfusion imaging with 3D segmentation to assess cerebral perfusion changes, and repeat neurological and neuropsychological evaluations using the same tests administered preoperatively and at discharge (NIHSS, mRS, MoCA, RAVLT, Kohs Block Design, and Stroop tests). The planning of the follow-up is describes in Table 2.

**Table 2** Inclusion and exclusion criteria for patient selection.

| **Investigation** |  | **Follow up assessments** | | | |  |
| --- | --- | --- | --- | --- | --- | --- |
|  | Before intervention | | After intervention | At 6 months | Unscheduled visit | |
| Baseline information | + | |  |  |  | |
| Informed consent | + | |  |  |  | |
| CTA | + | | + | + | + | |
| DSA | + | |  |  |  | |
| MRI ASL | + | | + | + |  | |
| Cognitive and neurologic status | + | | + | + | + | |
| Medication history | + | | + | + | + | |
| Ischemic events | + | | + | + | + | |

*Data processing*

All collected data will be meticulously entered into a database, where revascularization progress, the development of collateral circulation, and perfusion changes correlated with neurological and cognitive shifts will be evaluated. All data will undergo statistical processing and analysis to identify patterns and correlations, as well as survival analysis to assess collateral development and psychoneurological dynamics. All participants are expected to complete study visits over the planned 6-months follow-up period.

*Statistical analysis*

Data cleaning and coding will be performed using Microsoft Excel (Microsoft Office). Statistical analyses will be conducted using Stata statistical software, version 19 (StataCorp LLC, College Station, TX, USA). Descriptive analyses will summarize data using frequencies and percentages where appropriate. The distribution of continuous variables will be assessed using histograms and scatter plots. Continuous variables that follow a normal distribution will be reported as means ± standard deviations (SD), while categorical variables will be presented as percentages.

Inferential statistical tests will be selected based on variable type and data distribution. Associations between continuous and categorical variables will be evaluated using Student’s t-test, one-way analysis of variance (ANOVA), or the Wilcoxon signed-rank test, as appropriate. Categorical variables will be compared using the chi-square test. A two-sided p-value of <0.05 will be considered statistically significant.

Additional statistical analyses will be performed as appropriate to address specific research questions.

**Data Storage and Confidentiality**

The principles and standards of scientific ethics will be strictly observed throughout the study, including the prevention of data fabrication, falsification, plagiarism, and false authorship. Researchers will implement measures to protect participants’ personal information, including data coding and restricted access to identifiable data. The study will also be conducted in accordance with the principles outlined in the Declaration of Helsinki. Intellectual property rights will be governed by the legislation of the Republic of Kazakhstan and agreements among the study’s co-investigators. Key results will be disseminated through scientific articles, conference abstracts, posters, presentations, and databases. Intellectual property rights will be shared according to each researcher’s contribution. Filing for patents is not planned.

**V. Risk/Benefit Assessment**

**1. Level of risk:** Minimal.

**2. Potential risks:** The study does not pose any additional risks.

**3. Alternatives for participants:** Participants may withdraw from the study at any time.

**VI. Methods for Participant Selection.**

Patients with large ischemic lesions and brain atrophy confirmed by magnetic resonance imaging (MRI), as well as those with severe somatic conditions. These criteria will allow the study to focus on the most representative patient group, ensuring more reliable and well-founded results.

**Consent process:** Patients will only be able to participate in the study after providing informed consent.

**Participant condition:** All decisions regarding patient inclusion will be made by the researchers in accordance with the inclusion and exclusion criteria.

**Consent forms:** Available.

**Documentation of consent:** Available.

**Cost of participation:** None.

**Payment for participation:** None.
